# Dilated Cardiomyopathy and Later Onset Limb-Girdle Muscular Dystrophy Associated With *Fukutin* and *LaminA/C* Mutations

**DOI:** 10.1016/j.jaccas.2026.107038

**Published:** 2026-02-20

**Authors:** Alejandra Cardona Perez, Renee Moenning, Cynthia Bodkin, Laurie Gutmann, Benjamin M. Helm, Layne Wells, Steven A. Moore, Onyedika J. Ilonze

**Affiliations:** a Department of Internal Medicine, Indiana University School of Medicine, Indianapolis, Indiana, USA; b Department of Rheumatology, Indiana University School of Medicine, Indianapolis, Indiana, USA; c Department of Neurology, Indiana University School of Medicine, Indianapolis, Indiana, USA; d Department of Medical and Molecular Genetics, Indiana University School of Medicine, Indianapolis, Indiana, USA; e Department of Pathology, University of Iowa Carver College of Medicine, Iowa City, Iowa, USA; f Division of Cardiovascular Medicine, Krannert Cardiovascular Research Center, Indiana University, Indianapolis, Indiana, USA

**Keywords:** cardiac transplant, cardiomyopathy, chronic heart failure, genetic disorders

## Abstract

**Background:**

Nonischemic dilated cardiomyopathy (DCM) can result from pathogenic variants in genes affecting myocardial structure and function. *FKTN* and *LMNA* mutations may involve both cardiac and skeletal muscle, consistent with limb-girdle muscular dystrophy (LGMD), with cardiac disease sometimes preceding neuromuscular symptoms.

**Case Summary:**

We report on 2 adults presenting with advanced DCM requiring heart transplantation, who were later diagnosed with LGMD. A 22-year-old woman had biallelic *FKTN* variants, and a 37-year-old man carried a heterozygous *LMNA* pathogenic variant. Both had elevated creatine kinase prior to proximal muscle weakness. Muscle biopsy and genetic testing confirmed dystrophic processes.

**Discussion:**

These cases demonstrate that genetically mediated DCM may initially present as isolated cardiac disease. Early genetic testing can guide transplant planning, long-term care, and family counseling.

## Visual Summary

**Figure:**
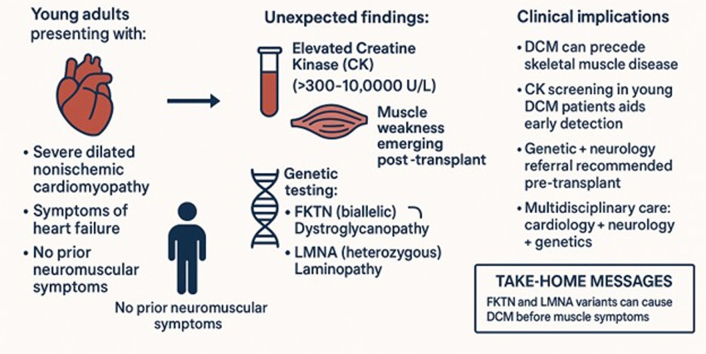

**Visual Summary:**
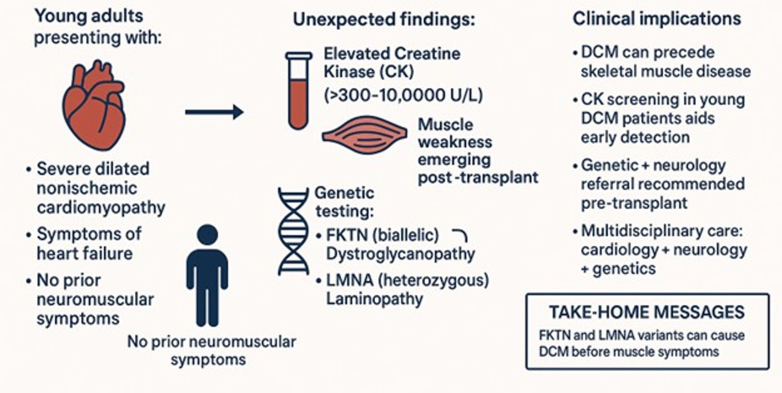
Dilated Cardiomyopathy and Later Onset Limb-Girdle Muscular Dystrophy Associated With *Fukutin* and *LaminA/C* Mutations Young adults with severe DCM due to *FKTN* and *LMNA* variants later developed skeletal muscle weakness. Elevated CK (>300-10,000 U/L) should prompt evaluation for muscular dystrophy and genetic testing. Early multidisciplinary collaboration supports accurate diagnosis, transplant planning, and lifelong care. CK = creatine kinase; DCM = dilated cardiomyopathy.

Nonischemic dilated cardiomyopathy (DCM) is characterized by left ventricular or biventricular chamber enlargement accompanied by a reduced left ventricular ejection fraction (LVEF) of <45%, in the absence of significant coronary artery disease. The true prevalence of DCM is likely underestimated and is influenced by geographic, ethnic, and methodological variation. A considerable proportion of cases have a genetic basis, with pathogenic variants in genes encoding structural and functional cellular proteins driving disease pathogenesis.^1^

**Take-Home Messages**

• Pathogenic variants in *FKTN*, *LMNA*, and related genes can cause dilated cardiomyopathy that precedes or overshadows neuromuscular symptoms, underscoring the importance of considering genetic etiologies early.
• Creatine kinase measurement can serve as a low-cost, preliminary screening tool, but genetic testing remains the cornerstone for identifying dystrophic processes.
• Routine genetic counseling and testing should be considered in all patients with nonischemic dilated cardiomyopathy to uncover underlying hereditary disorders that may affect management, lifelong surveillance, and genetic family counseling.

Genetic forms of DCM may have extracardiac involvement and manifestations, including neuromuscular disorders such as limb-girdle muscular dystrophies (LGMDs). LGMDs encompass a clinically and genetically heterogeneous group of inherited myopathies characterized by progressive weakness of the pelvic, shoulder girdle, and proximal extremity muscles, with variable cardiac involvement. In severe cases, cardiac involvement in LGMD, including conduction abnormalities, arrhythmias, and DCM, may progress to end-stage heart failure (HF). Therefore, establishing a genetic etiology is critical for guiding management, prognostication, and family counseling.^2^^,^^3^

Recognition of neuromuscular disease in patients primarily presenting with DCM remains challenging, given the wide variability in clinical phenotypes, even within a genetic subtype. This is particularly difficult when overt skeletal muscle symptoms are absent or delayed. Identifying the genetic cause of neuromuscular disease, such as LGMD, manifesting after a diagnosis of DCM is rare, yet recognition is critical, as it has implications for heart transplantation candidacy, postoperative management, and long-term neuromuscular care.^4^^,^^5^

In this report, we describe 2 adults with DCM who progressed to end-stage HF requiring heart transplantation. Both were later found to have elevated creatine kinase (CK) of unclear origin, prompting further evaluation that led to the diagnosis of LGMD. These cases highlight the importance of comprehensive evaluation in patients with nonischemic cardiomyopathy and support the incorporation of genetic counseling and testing into routine clinical care.

## Case Presentations

### Case 1

A 22-year-old woman presented with 10 months of abdominal pain and early satiety, leading to multiple hospitalizations and extensive but unrevealing evaluations. Over time, she developed symptoms of HF with evidence of end-organ dysfunction, prompting a cardiovascular assessment. Transthoracic echocardiography revealed an LVEF of 10%, moderately dilated left ventricle (LV), severe mitral regurgitation and tricuspid regurgitation, and reduced right ventricular function. The patient denied any family history of cardiac diagnoses, cardiomyopathy, sudden or unexplained deaths, arrhythmia, neuromuscular disease, or history of genetic testing. Cardiac magnetic resonance imaging demonstrated prominent LV trabeculations concerning for LV noncompaction.

**Figure 1:**
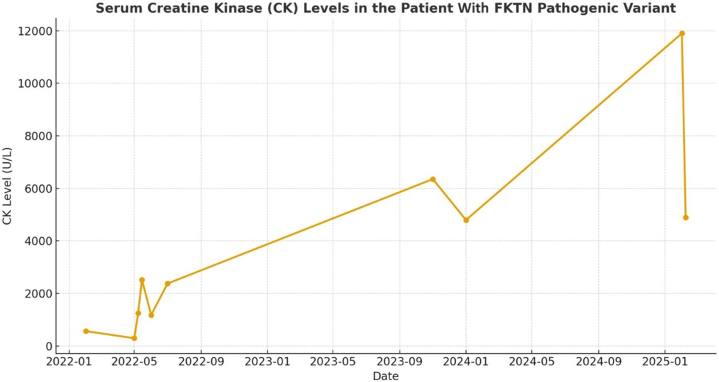
Creatine Kinase Levels in the Patient With Pathogenic Variants in *FKTN* (Case 1) Serial CK measurements demonstrate persistently elevated levels ranging from 298 to 11,900 U/L over 3 years. The progressive rise in CK preceded the onset of skeletal muscle weakness and ultimately supported the diagnosis of limb-girdle muscular dystrophy associated with pathogenic *FKTN* variants. CK = creatine kinase.

**Figure 2:**
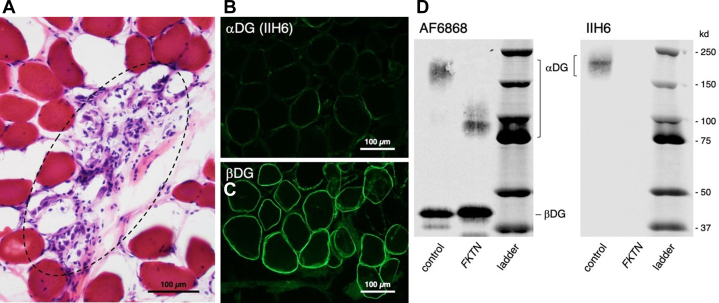
Muscle Biopsy Evaluation for the Patient With Pathogenic Variants in *FKTN* (Case 1) Muscle biopsy evaluation is diagnostic of dystroglycanopathy. Frozen sections from the *FKTN* patient's muscle biopsy were evaluated by (A) H&E, (B and C) immunofluorescence, and (D) Western blotting. Grouped muscle fibers are undergoing myonecrosis with active myophagocytosis (black dashed oval). Sarcolemmal staining for α-dystroglycan (αDG) with the matriglycan-specific antibody IIH6 is greatly reduced, while sarcolemmal staining for β-dystroglycan (βDG) is normal. Glycoproteins are enriched in muscle biopsy homogenates using affinity binding to wheat germ agglutinin-coated beads. After electrophoresis on gradient gels, membranes are blotted with AF6868 to evaluate the molecular weights of αDG and βDG or with IIH6 to evaluate matriglycan. In the *FKTN* biopsy sample, the size of αDG is greatly reduced and does not bind IIH6. H&E = hematoxylin and eosin.

The patient was initiated on maximum tolerated HF pharmacotherapy. However, she remained symptomatic (NYHA functional class IIIB) and was hospitalized for evaluation of heart transplantation. Her condition significantly deteriorated, and she ultimately received a heart transplant. Histopathology of the native heart demonstrated increased trabeculation and patchy interstitial fibrosis. Serial endomyocardial biopsies showed low-grade inflammation without significant rejection. CK levels were found to be elevated (567 U/L); statin therapy was discontinued, and CK was monitored during routine follow-ups. CK progressively increased, as seen in Figure 1, peaking at 11,900 U/L, and at 2 years post-transplant, she developed progressive proximal muscle weakness. Electromyography revealed myopathic changes, and an extended myositis panel was low-positive for PM/Scl-100 antibody. She had no clinical findings consistent with connective tissue disease. Autoantibodies associated with systemic rheumatic disease, including antinuclear antibody, anti–cyclic citrullinated peptide, anti–double-stranded DNA, anti-Ro, anti-La, anti-Jo-1, anti-Smith, ribonucleoprotein, beta 2-glycoprotein, anti-HMGCR, SCL-70, cardiolipin, and rheumatoid factor, were negative. Biceps femoris biopsy demonstrated mild necrotizing myopathy, suggestive of muscular dystrophy. Genetic testing identified biallelic novel variants in the *FKTN* gene: a splice-site pathogenic variant (intron 10, c.1172+1G>A, heterozygous) and a novel missense variant (exon 10, c.1135A>G; p.Asn379Asp, heterozygous). The latter was initially classified as a variant of uncertain significance but was computationally predicted to be disruptive, and follow-up studies confirmed its pathogenic effect. Given these variants, a muscle biopsy was performed to evaluate α-dystroglycan. Histopathology, immunofluorescence, and Western blotting confirmed the diagnosis of dystroglycanopathy (Figure 2). These studies were critical to confirm the pathogenicity of the novel missense variant.

### Case 2

A 37-year-old man with chronic atrial and ventricular tachycardias was first diagnosed with DCM at age 22 and remained asymptomatic from a HF perspective (NYHA functional class I). Transthoracic echocardiography on initial evaluation showed an LVEF of 37% with global wall motion abnormalities. Cardiac magnetic resonance imaging showed LV dilatation and reduced systolic function, as well as akinetic wall motion abnormality involving the lateral wall and apex of the LV.

The patient was initiated on HF and antiarrhythmic therapies and eventually underwent implantable cardioverter-defibrillator (ICD) placement. Despite optimizing his pharmacological therapy, he continued to experience frequent ICD shocks, and heart transplantation was recommended, which he received at age 35. Native heart biopsy showed focal areas of fibrosis, fatty infiltration, myocyte hypertrophy, and ventricular dilatation, consistent with DCM. During his pretransplant work-up, it was noted on physical therapy evaluation that his lower extremities were weak, attributed to the severity of his HF. At 1 year post-transplant, he noted difficulty with gait and rising from a chair; symptoms that progressed after heart transplant. On examination, he demonstrated lower extremity weakness localized to the hip girdle. CK was 821 U/L. Statin therapy was discontinued, and a recheck months later showed CK of 1024 U/L. He was referred to the neuromuscular clinic for further evaluation and underwent genetic testing that revealed a pathogenic variant in the *LMNA* gene (exon 10, c.1622G>A; p.Arg541His). Additionally, 2 variants of uncertain significance were identified: *BAG3* (c.160G>C; p.Val54Leu) and *CACNA1C* (c.5098G>A; p.Gly1700Ser). There was no family history of cardiac or neuromuscular disease.

## Discussion

This case series underlines the intersection between DCM and later onset LGMD due to pathogenic variants in the *FKTN* and *LMNA* genes. Both patients required heart transplantation and demonstrated progressive skeletal muscle manifestations consistent with dystrophic processes, along with a persistently elevated CK (see Table 1 for a comparison of cases). These findings illustrate how cardiomyopathy may precede or overshadow neuromuscular symptoms, complicating early recognition of an underlying muscular dystrophy.

**Table 1:** Comparative Summary of Cases 1 and 2

	Case 1	Case 2
Age/Sex	22-year-old woman	37-year-old man
Initial Presentation	Abdominal pain, decreased appetite, PND, orthopnea, progressive dyspnea, peripheral edema	Palpitations, narrow complex tachycardia >200 beats/min
Initial cardiac findings	TTE: LVEF 10%-15%, moderately dilated left ventricle, severe eccentric MR, moderate TR, reduced right ventricle function CMR: left ventricle noncompaction, reduced left ventricular systolic function	TTE: LVEF 37% with global wall motion abnormalities CMR: left ventricle dilation, reduced systolic function, akinetic lateral wall and apex
Genetic findings	Two pathogenic *FKTN* variants (splice-site + novel missense with pathogenicity confirmed by α-dystroglycan studies)	Pathogenic *LMNA* variant (c.1622G>A); variant of undetermined significance in *BAG3* (c.160G>C) and *CACNA1C* (c.5098G>A)
Management before OHT	GDMT (losartan, torsemide)	GDMT (sacubitril-valsartan, spironolactone, metoprolol succinate), mexiletine; ICD and multiple ablations for arrhythmias
Heart failure severity	NYHA functional class III	NYHA functional class I
Indication for OHT	Progressive HF despite GDMT	Refractory arrhythmias and frequent ICD shocks
Age at OHT	23 y	35 y
Native heart pathology	Increased trabeculation, patchy interstitial fibrosis	Focal fibrosis, fatty infiltration, myocyte hypertrophy, left ventricular dilatation
Post-OHT course	Initial NYHA functional class I post-transplant. Found to have persistently elevated CK (1,000-2,000 U/L). At 2 years: severe myopathy, CK peak 11,900 U/L, EMG myopathic changes, biceps biopsy nonspecific; dystroglycanopathy confirmed by α-dystroglycan testing	After 1 year, patient developed progressive proximal lower extremity weakness, CK of 821-1024 U/L; statin discontinued, CK remained elevated; neuromuscular evaluation confirmed *LMNA*-related myopathy

CK = creatine kinase; CMR = cardiac magnetic resonance; EMG = electromyography; GDMT = guideline-directed medical therapy; HF = heart failure; ICD = implantable cardioverter-defibrillator; LVEF = left ventricular ejection fraction; MR = mitral regurgitation; OHT = orthotopic heart transplant; PND = paroxysmal nocturnal dyspnea; TTE = transthoracic echocardiography; TR = tricuspid regurgitation.

Pathogenic variants in *FKTN* can disrupt glycosylation of α-dystroglycan, impairing muscle membrane stability, and can manifest as DCM before skeletal muscle symptoms occur.^6^ In contrast, *LMNA*-related disease arises from alterations in nuclear envelope structure and signaling, leading to both skeletal and cardiac involvement. Cardiac manifestations in *LMNA*-associated disease most commonly include conduction system disease and malignant arrhythmias, although progressive ventricular dysfunction and DCM are also well recognized.^4^^,^^5^^,^^7^ Consistent with prior reports, our cases underscore the phenotypic heterogeneity of these genotypes and reaffirms that cardiac disease may be the presenting feature even in conditions traditionally associated with neuromuscular involvement.^1^

CK is a hallmark biochemical feature of muscular dystrophy with high sensitivity, and it may serve as a useful, low-cost screening tool in those with DCM.^8^ Markedly elevated CK (>1000 IU/L) is strongly associated with myopathies, including LGMD, but it cannot distinguish between subtypes or other myopathies.^9^ Definitive classification requires molecular genetic testing, which not only establishes a precise diagnosis but also informs prognostication and risk stratification.

Beyond *FKTN* and *LMNA*, other genes have been associated with LGMD and cardiomyopathy phenotypes, including Fukutin-related protein (*FKRP*). Similar to our patients, individuals with *FKRP*-related disease may present initially with severe cardiovascular disease, elevated CK levels, and mildly progressive muscular dystrophy.^4^^,^^8^^,^^10^ In addition to LGMD, there are other muscular dystrophies diagnosed in adulthood that are associated with structural and electrical cardiovascular manifestations, which can eventually lead to HF. These may also present in an isolated nature or may supersede neuromuscular symptoms.^1^^,^^4^

Given this overlap, cardiologists may be the first specialists to evaluate these patients after being referred for asymptomatic LV dysfunction or elevated biomarkers such as CK, troponin, and B-type natriuretic peptide, suggestive of myocardial injury.^11^ This emphasizes the importance of maintaining a high index of suspicion for underlying neuromuscular disease in younger patients presenting with DCM, particularly when accompanied by persistently elevated CK levels (>300 IU/L). Identifying a dystrophic process early in its course supports targeted genetic counseling and optimizes post-transplant and long-term multidisciplinary care.

Based on these observations, genetic counseling and testing should be considered in all patients with nonischemic cardiomyopathy, with CK measurement serving as a screening tool, particularly in younger patients (≤45 years) with DCM. If a dystrophic process is suspected or confirmed, early interdisciplinary collaboration among cardiology, neurology, and genetics specialists is essential to guide care and longitudinal surveillance.

## Conclusions

This case series demonstrates that genetically mediated DCM due to *LMNA* and *FKTN* variants can initially manifest with isolated cardiac disease, with skeletal muscle involvement emerging later in the disease course after heart transplantation. While persistently elevated CK measurement can provide an important diagnostic clue, molecular genetic testing is essential to establishing etiology, informing prognosis, and guiding family risk assessment.

Given the phenotypic heterogeneity and potential for early cardiac involvement, these findings strongly support the integration of genetic counseling and testing into routine evaluation of all patients with nonischemic DCM, particularly younger individuals or those with unexplained elevated CK. Early identification of a pathogenic variant allows for multidisciplinary management, genetic counseling, and informed decision-making regarding advanced therapies. Prioritizing genetic evaluation in DCM bridges the gap between cardiac care and neuromuscular disease recognition, improving diagnostic accuracy and patient outcomes.

## Funding Support and Author Disclosures

Dr Moore is partially supported by the National Institute of Neurological Disorders and Stroke of the National Institutes of Health through the Iowa Wellstone Muscular Dystrophy Specialized Research Center (P50NS053672). Dr Ilonze is supported by the Winn Excellence in Clinical Trials Career Development Award. The content is solely the responsibility of the authors and does not necessarily represent the official views of the National Institutes of Health. All other authors have reported that they have no relationships relevant to the contents of this paper to disclose.
